# Anesthesiologists With Advanced Degrees in Education: Qualitative Study of a Changing Paradigm

**DOI:** 10.2196/38050

**Published:** 2022-06-30

**Authors:** Anuj Aggarwal, Olivia Hess, Justin L Lockman, Lauren Smith, Mitchell Stevens, Janine Bruce, Thomas Caruso

**Affiliations:** 1 Department of Anesthesiology, Perioperative, and Pain Medicine Stanford University School of Medicine Stanford, CA United States; 2 Stanford University School of Medicine Stanford, CA United States; 3 Department of Anesthesiology and Critical Care Medicine Children’s Hospital of Philadelphia Perelman School of Medicine at the University of Pennsylvania Philadelphia, PA United States; 4 Graduate School of Education Stanford University Stanford, CA United States; 5 Department of Pediatrics Stanford University School of Medicine Stanford, CA United States

**Keywords:** academic medical centers, trends, medical education, medical, faculty, anesthesiologists, medical professionals, learning, institute, clinician, educator, experience, decision-making, training

## Abstract

**Background:**

Anesthesiology education has undergone profound changes over the past century, from a pure clinical apprenticeship to novel comprehensive curricula based on andragogic learning theories. Combined with institutional and regulatory requirements, these new curricula have propagated professionalization of the clinician-educator role. A significant number of clinician-educator anesthesiologists, often with support from department chairs, pursue formal health professions education (HPE) training, yet there are no published data demonstrating the benefits or costs of these degrees to educational leaders.

**Objective:**

This study aims to collect the experiences of anesthesiologists who have pursued HPE degrees to understand the advantages and costs of HPE degrees to anesthesiologists.

**Methods:**

Investigators performed a qualitative study of anesthesiologists with HPE degrees working at academic medical centers. Interviews were thematically analyzed via an iterative process. They were coded using a team-based approach, and representative themes and exemplary quotations were identified.

**Results:**

Seven anesthesiologists were interviewed, representing diverse geographic regions, subspecialties, and medical institutions. Analyses of interview transcripts resulted in the following 6 core themes: outcomes, extrinsic motivators, intrinsic motivators, investment, experience, and recommendations. The interviewees noted the advantages of HPE training for those wishing to pursue leadership or scholarship in medical education; however, they also noted the costs and investment of time in addition to preexisting commitments. The interviewees also highlighted the issues faculty and chairs might consider for the optimal timing of HPE training.

**Conclusions:**

There are numerous professional and personal benefits to pursuing HPE degrees for faculty interested in education leadership or scholarship. Making an informed decision to pursue HPE training can be challenging when considering the competing pressures of clinical work and personal obligations. The experiences of the interviewed anesthesiologists offer direction to future anesthesiologists and chairs in their decision-making process of whether and when to pursue HPE training.

## Introduction

Since the advent of anesthesiology, a combination of societal, economic, and higher education movements has influenced the adoption of formal learning theories by anesthesiology educators [1]. Initially, anesthesiology education centered around a clinical apprenticeship. However, the advancement of surgical techniques and the drive for reliable anesthetic techniques resulted in the restructuring of anesthesiology programs over the second half of the twentieth century [2,3]. Although pedagogical learning theories originally dominated anesthesiology training, the end of the last century marked the introduction of andragogic and experiential learning theories that accompanied the expansion of formal curricula for anesthesiology trainees (Table 1) [4].

These new curricula, combined with expanding institutional and regulatory requirements, have propagated a professionalization of a clinician-educator role for anesthesiologists. Many academic physicians have transitioned from the traditional clinician-researcher-educator role, and instead pursue educational scholarship as the central facet of their academic careers [5]. In the United States, this evolution has been influenced by the Accreditation Council for Graduate Medical Education (ACGME), which states that graduate medical education program directors should have at least 3 years of educational or administrative experience [6]. Though this statement by the ACGME calls for a broad allowance of education and administrative experiences, department leadership have recognized the value of formal training in education. Over the past several decades, this environment has propagated the development of health professions education (HPE) programs that are specifically designed for physicians, including certificate, master, and doctoral programs. There are now more than 150 such programs worldwide, with significant variation in content delivery (online, asynchronous, hybrid, or in-person), duration, and curriculum [7-9].

Additionally, there has recently been an emphasis on blended training environments that incorporate nonclinical interests, physician wellness, and social justice [1]. This progression of learning paradigms is also contributing to the increased growth of HPE programs [8,10]. Despite the rising number of anesthesiologists seeking HPE, there are limited data demonstrating the benefits and costs of these programs [9]. Many anesthesiologists who are interested in education-oriented careers are without appropriate guidance about whether to pursue an HPE degree. This is further compounded by the variety of programs, ranging from certification programs at local institutions to formal degree–granting programs.

There is an emerging community of anesthesiologists who have attained HPE degrees, along with others who are in the process of completing HPE programs, which grant master’s degrees or higher. This qualitative study investigates the experiences of anesthesiology educators who have completed HPE master’s degrees. It seeks to understand the influence these degrees have had on their professional and personal advancement and demonstrate common, valuable elements for future anesthesiologists wishing to pursue HPE programs.

**Table 1:** Examples of andragogy and pedagogy learning styles.

Andragogy	Pedagogy
Self-directed learner	Teacher-dependent learner
Greater life experiences	Little to no life experience
Learning determined by social roles	Learning determined by teacher
Problem centered	Content centered
Intrinsic motivation	Extrinsic motivation

## Methods

### Study Design and Research Characteristics

We performed a prospective, semistructured interview qualitative study with thematic analysis. The study design and reporting adhere to the standards described by the Standard for Reporting Qualitative Research guidelines [11]. All participants were approached via direct, electronic solicitation and were informed that participation was voluntary. Study participation offered no direct benefit to the participants. The interviewers obtained verbal consent from all study participants.

### Context

Investigators interviewed participating anesthesiologists who worked at academic medical centers representing different regions of the country and different anesthesiology subspecialties. All interviews were conducted remotely using video conference software (Zoom Video Communications) to maintain safe social distancing, as interviews were performed during the COVID-19 pandemic. The interviews occurred from August through October of 2020.

### Sampling Strategy

The study team sought to collect a sample of up to 12 anesthesiologists or until thematic saturation occurred. Participants were identified based on national reputation and professional relationships representing different institutions and different subspecialities [12]. Nonprobability sampling is typical in qualitative research, where the goal is not to randomly select from the population, but rather to purposefully identify and select relevant individuals [13]. This homogenous group sampling provides a rich understanding of their experiences [13]. We selectively recruited academic anesthesiologists who had earned a master’s degrees in HPE. During qualitative research, data collection and analysis often occur simultaneously [13]. Sample size is considered adequate when little additional information is emerging from the interviews, which began at the 6th interview, resulting in a total of 7 participants.

### Ethics Approval

The Stanford University Institutional Review Board granted a waiver for this study (Protocol #57512).

### Data Collection Methods

Prior to each interview, we collected demographic data via questionnaires administered electronically using Research Electronic Data Capture [14,15]. Interviews were audio recorded, and transcripts were subsequently generated using an automated audio transcription service and were reviewed for accuracy prior to analysis (Otter.ai).

### Data Collection Instruments

Via the preinterview survey instrument, the participants provided us with information regarding the duration of the HPE program they completed, time since completion of the program, degree granted by the program, granting institution, the format of educational content delivery, and the tuition funding sources. Subsequent interviews used a semistructured interview guide, which remained unchanged (Multimedia Appendix 1). The interview guide consists of 7 open-ended questions accompanied by 8 optional probing questions and prompts to increase the amount of information provided by each participant for a given question (Multimedia Appendix 1). Two members of the study team (JB and TC) with expertise in qualitative methodology designed the interview guide. The questions addressed motivation, timing (stage of career), perceptions of the educational program, perception of barriers to completing the program, and summative or wholistic opinions of the program. To maintain consistency of the interview technique, the same study investigator (AA) conducted all interviews.

### Data Analysis

Interview transcripts were analyzed using an iterative, grounded theory approach to identify common themes [16,17]. Each theme was defined using a representative quotation from an interview. In cases where investigators coding the transcripts disagreed about the categorization of themes, a third investigator served as arbitrator. Because we were more interested in the relative importance and connection of ideas compared to the frequency of ideas, we performed this inductive thematic analysis, not a content analysis [13]. After the analysis, we presented the results to 2 of the participants to member check, which provided confirmation that the thematic analysis aligned with the message they sought to impart [13].

## Results

### Participants

All anesthesiologists approached for the study agreed to participate, for a total of 7 physician participants representing 6 different academic medical centers and representing the ranks of assistant and associate professor (Table 2). By self-identified gender, 4 (57%) of the participants were female and 3 (43%) were male. In total, participant degrees included 4 different HPE degrees granted by 6 different academic institutions; all participants completed a master’s degree. Moreover, 2/7 (29%) participants reported completing HPE training prior to attending medical school, and the remaining after their anesthesiology graduate medical education training. The 2 participants who completed degrees prior to medical school were self-funded for tuition and expenses, and department resources funded tuition of the 5 who earned HPE degrees after joining a faculty as academic physicians. The most recent HPE graduate completed their program 5 years ago; the most distant graduate was 25 years ago. For participants who completed their HPE degrees after completing residency or fellowship training, the range of completion date was 5-9 years ago. The median time since the receipt of HPE among the 7 participants was 6 years. Time to programmatic completion ranged from 1 to 6 years, with a median of 2 years. HPE instructional formats included in-person, virtual, and hybrid. The interviews lasted between 18 and 28 minutes. After the 7 interviews with iterative analyses were conducted, little additional information emerged, and it became apparent that thematic saturation had occurred.

**Table 2:** Summary of participant demographics and experiences.

Variables			Values
Time since degree completion (years), mean (range)			10.4 (5-25)
Duration of degree program (years), mean (range)			2.71 (1-6)
**Degrees completed by interviewees, n (%)**			
	Master of Education	4 (57)	
	Master of Education in the Health Professions	1 (14)	
	Master of Science in Health Professions Education	1 (14)	
	Master of Academic Medicine	1 (14)	
**Degree-granting institutions, n (%)**			
	Harvard	2 (29)	
	Johns Hopkins	1 (14)	
	Massachusetts General Hospital	1 (14)	
	University of Cincinnati	1 (14)	
	University of Houston	1 (14)	
	University of Southern California	1 (14)	
**Self-identified gender of interviewees, n (%)**			
	Female	4 (57)	
	Male	3 (43)	
**National regions represented by interviewees, n (%)**			
	West Coast	2 (29)	
	Northeast	3 (43)	
	South	1 (14)	
	Upper Midwest	1 (14)	
**Tuition funding source, n (%)**			
	Departmental	5 (71)	
	Self-funded	2 (29)	

### Thematic Results

Our analysis of interview transcripts identified the following 6 core themes: outcomes, extrinsic motivators, intrinsic motivators, investment, experience, and recommendations (Table 3).

**Table 3:** Representative statements from the thematic analysis.

Code	Definition	Representative statements
Outcomes	Intended and unintended impacts of the degree on one’s career, including how they have used the degree and practical day-to-day application of skills or knowledge	“It’s definitely a factor to get you noticed by people like chairs when they have educational leadership roles to fill.” “I think it has been a good opportunity for me to… further push those key projects…[and] get a better understanding of where the problems are in care delivery within my department.” “I think it influenced much of what I did even beyond education, when I look back, what really happened in the years just after, it just gave me that desire to know my own self, to just go for it. And that was really great to develop more confidence in your own abilities outside of what you do.” “I mean, like the job offers I’m getting, it’s insane.”
Extrinsic motivators	Reasons for an individual seeking a degree based on attaining a known, external reward	“Pretty much… [the chair] told me I had to do it to become faculty.” “I would say that [for] the department and my division, definitely, it was an expectation that I would pursue the degree.” “… my career goals were to kind of move up [to] med ed administration and to publish in medical education.” “My mentor… was a very key proponent in me getting my masters because… to continue to move up that that would be a skill set and a degree that would look good from an experience standpoint.”
Intrinsic motivators	Reasons for an individual seeking a degree for its own sake without an external reward, including emotions, values, and goals	“I was just frustrated with myself. And I felt like I just needed a formalized process and I needed everything at once and I was tired of trying to find it on my own.” “I felt like I needed to know the language and I needed to know the theory behind why things are done the way they're done in medical education. And so that prompted me to get my masters.” “I really wanted advanced training and knowledge in education in general, which I thought would be helpful, just to understand more what's going on” “I started to really become interested in studying educational processes, and team dynamics even, and the ways we think and how it influences the way we act and just everything like that.”
Investment	Positive and negative aspects of obtaining an HPE^a^ degree, including personal or financial sacrifices, opportunity costs, and time commitment	“The biggest stressor was that I had to negotiate with my family because of time.” “I didn't jump into the program my first year as an attending even though I was advised to, because I felt like I really needed to lay my ground as a clinician. … We work a lot of days in a row. And that makes doing an online curriculum while you're a full-time employee very difficult…” “When I enrolled in the program, I had the added pressure to really get through it as fast as possible… because there was this tension with my family, basically.” “First of all, it's a time commitment. …If you just stay in your clinical practice, right, and you try to do things within the division or department, it's already very busy.”
Experience	Overall perspectives about the degree program, including opinions about the process of obtaining the degree (ie, satisfaction with the content covered, mode or format of delivery, and suggestions for improvement)	“It reviews a lot of the scientific methodology that we all appreciate even in other aspects of research. There's an emphasis on leadership, which I really appreciated. I especially appreciated that understanding of ourselves. There was an emphasis on understanding your MBTI scores and what that meant, which really gets into where you understand your strengths, and what works well.” “But what I wish the program did was potentially focus less on individualized projects and potentially allow more collaboration and group projects for your Capstone… It would be really interesting to use the program more to develop interprofessional projects than having everybody do one individual project.” “I think what I really would have loved is if there was somebody in there who could help you either write a case report, you know, or help you with the research part as you're doing it, or help you write a grant.”
Recommendations	Advice that the participant would offer to someone interested in pursuing an HPE degree regarding timing, factors to consider, and aspects of a program to look for or avoid	“If you think, look, I love to teach… you don't need a master's degree to be a teacher of residents, right, anybody in an academic center is going to teach residents. But if you think you want to be involved in residency leadership or medical school leadership, if you see yourself as being a program director one day or you know, dean for curriculum of a medical school, that kind of thing, then I think it is a good step because as I said before, I think it will get you noticed when those kinds of opportunities come up.” “I think right out of training, you don't necessarily know which person you are. What I usually advise is to do some workshops, figure out if you just want to become a really good teacher. I think you don't need a master’s to do that.” “I think if you want to study teaching and you want to have a foundation in adult learning theory and you want to be able to become an administrator or become a researcher in med[ical] ed[ucation] that I would advise the [HPE degree].” “If you're going to use this degree, you're pretty much marrying yourself to academics. But then I would also say that I think that there's a lot of opportunities for innovation, and a lot of interesting ways to use the master of education. And I would also say that I would sort of make sure that I had an academic or administrative niche that, you know, you can really start applying the coursework early on. So that you know, like, you can sort of build your academic portfolio while you're working on the degree.”

^a^HPE: health professions education.

#### Outcomes

The participants consistently highlighted intended and unintended consequences from formal HPE training. Among the intended outcomes, the participants noted that the training prepared them for educational leadership roles within their departments and enhanced recognition from other departmental leaders. They also reported being better equipped to augment their daily academic activities, including learner assessment, clinical teaching improvement, and production of scholarship. Unintended but positive outcomes included earlier promotion and offers from other institutions for specific education appointments. The participants also noted a theme of self-exploration and discovery from their HPE experiences.

#### Extrinsic Motivators

The participants highlighted 3 recurring extrinsic motivating factors for pursuing formal HPE training, which were as follows: expectations from departmental leadership, availability of financial support, and opportunities for career advancement. Moreover, 4/7 (57%) participants noted that the completion of formal HPE programs allowed them to fulfill departmental requirements for promotion and to work with specific mentors in a structured way. Those who completed HPE programs after beginning their careers noted that financial tuition support served as a positive motivator.

#### Intrinsic Motivators

Two common intrinsic motivators for pursuing formal HPE training emerged, which were (1) a passion for education and teaching and (2) personal insight about a lack of knowledge of medical education theory. The participants sought to better understand the latest methodologies of medical education and noted feeling this was needed for career advancement along a medical education path; 1 (14%) participant said, “I needed to know the language and…the theory behind why things are done the way they’re done in medical education.” Moreover, 4 (57%) participants stated that their intrinsic motivation to pursue HPE training increased after becoming faculty (as gaps in their knowledge of education theory became clearer) compared to their motivation prior to becoming faculty.

#### Experience

The interviewees highlighted 3 experiential components of the HPE programs, which were structure, coursework, and research. Regarding structure, the participants noted that those entering HPE training programs should reflect on their own personal learning style to direct whether in-person, virtual, or hybrid learning best suits them. Programmatic flexibility was also important to the participants; they noted that given the complex schedules, asynchronous coursework helped them integrate varying clinical and administrative duties. The 5 (71%) participants who completed HPE programs after starting their careers all continued to work either full- or part-time while obtaining the degrees. Some commented that a more structured format was important to keep them focused, whereas others enjoyed the flexibility of completing coursework at their own pace.

Regarding coursework, the participants noted that the content complemented their personal and academic interests and enhanced their leadership abilities; in particular, content on leadership and professional development was highly regarded. Interdisciplinary programs that included professionals from other specialties, such as nursing, were viewed positively. The participants voiced some frustration around mandatory coursework that they perceived to be irrelevant to their goals, and regarding coursework that required a significant time investment. Additionally, 5 (71%) participants reported that they expected more statistical training, and at times, lack of statistical expertise may have hindered the completion of research projects. Nonetheless, the participants did endorse that they gained a better understanding of medical education research methodologies through the programs.

#### Investment

Common among interviews was the invested time and opportunity cost of HPE training programs. The participants specifically highlighted the balance between family, work, and programmatic demands as potential barriers when choosing to pursue an HPE program; 5 (71%) participants reported weighing the trade-off between establishing themselves as clinicians in their departments and the time necessary to dedicate to the educational program. Several participants (4/5, 80%) chose to delay starting HPE programs for several years after becoming faculty in order to establish clinical skills first. The participants noted that these programs led to an increase in “off hour” work and required a significant investment of time, often more than anticipated. Those participants (5/5, 100%) who pursued degrees after joining an anesthesiology department all received financial support for tuition.

#### Recommendations

The participants offered several key recommendations for colleagues considering pursuing formal HPE training. The first focused on program structure, with encouragements for colleagues to carefully evaluate the balance between virtual and in-person formats, the flexibility of course sequence, and time to degree completion; 3 (43%) recommended that individuals seek programs associated with their home institution for optimal flexibility. Regarding earning a formal HPE degree versus a teaching certificate, the participants recommended that individuals reflect on their motivation for pursuing additional training. Exemplary questions would probe, “Why pursue further education?” “What is the long-term goal?” “How will the training be utilized personally and professionally?” “Is there aspiration for an education leadership position that might be enhanced and more likely achieved by the completion of an HPE degree?”

Beyond career advancement, the participants highlighted that a genuine interest in education itself was important to ensure that the coursework is enjoyable. They suggested taking advantage of workshops or shorter programs prior to HPE enrollment to better understand whether pursuing further training might be enjoyable. Many (6/8, 75%) participants noted that having an HPE degree is not a prerequisite to being a talented educator, but the knowledge obtained from an HPE degree can be useful for learning methodologies for academic scholarship. There were different opinions regarding how to manage time commitments between work and HPE training. A common theme was the importance of practicing self-compassion given the time commitments and added stress of pursuing HPE training.

## Discussion

### Principal Findings

This qualitative study explored opinions of anesthesiologist educators who have completed formal HPE programs to provide a resource for physicians considering similar degrees. Recurring themes included outcomes on career, extrinsic and intrinsic motivators, personal investment, experience of obtaining formalized training, and recommendations. The participants noted that formal HPE training prepared them for leadership roles and to effectively engage in medical education scholarship. Further, they noted that the degree attracted the attention of other anesthesiology leaders seeking individuals for education leadership roles. They reported significant time investment, opportunity cost, and tension between personal and family well-being while training. The participants had insightful advice to guide anesthesiologists who are considering pursuing this degree, including stepwise introduction into the field of medical education, understanding motivations toward pursuing an advanced degree, and the recognition that being an educator and part of the medical education community does not necessarily require an advanced education degree.

This study provides future anesthesiology education leaders with guidance from several formal educators from around the United States. Faculty at departments where there are no anesthesiologists who have completed HPE training lack the opportunity to gain insights from colleagues when considering such training. Given the time and resources required to complete the programs, this information may help these individuals balance multiple personal and professional factors when deciding whether to pursue formalized educational training.

We suspect that the recent increased interest of anesthesiologists in pursuing HPE has emerged partly due to increasing ACGME expectations for faculty to not only administer training programs but also be educational innovators. Twenty-first century program directors must be capable of designing and implementing curricula while evaluating trainees using novel tools. Most medical school or anesthesiology residency programs do not equip physicians with expertise in learning theory, curriculum design, or evaluation and assessment [18]. HPE programs help faculty grow professionally, acquire knowledge, and join a community of like-minded individuals [19]. Further, these programs increase the quality of educational research by both subjective and objective measures [20]. Given the potential benefits to faculty and to anesthesiology trainees, the experiences gathered via the interviewees offer insights, caution, and encouragement for those considering HPE training. These themes may be particularly useful for those who lack institutional mentorship in 1 of the more than 160 anesthesiology residencies in the United States alone.

### Limitations

There were several limitations to this study. Given that this was a homogenous group sample, there is potential for sampling bias. In order to minimize bias, we sought anesthesiologists from different subspecialties, genders, and races. Further bias mitigation strategies included using 2 investigators who independently analyzed the interview transcripts and member checking after the analysis [13]. When considering gathering quantitative survey data from a larger cohort of anesthesiologists compared to this smaller sample of in-depth interviews, we chose the latter in order to gain deeper insight into each individual participant’s experience. A national survey of physicians with HPE degrees will further enrich the themes presented and is an area of future research. Further, the participants represented large academic medical centers, and the applicability of their experiences to medium or smaller sized anesthesiology departments or private practice groups is unclear. Lastly, since all participants completed their HPE programs in the past, recall bias may play a role in the answers the participants provided throughout the interviews.

### Conclusion

Our work offers insights from anesthesiology educators who have completed formal HPE training programs and can serve as a starting point for conversations for anesthesiologists who are considering pursuing similar programs. Future inquiries include larger survey data as well as longitudinal studies to observe career trajectories of individuals who pursued HPE degrees. The results support the benefits of HPE degrees for those who seek careers in medical education, especially those dedicated to pursuing careers in education leadership and scholarship.

## Appendix

Multimedia Appendix 1 Study interview questions.

## Abbreviations

ACGME: Accreditation Council for Graduate Medical Education
HPE: health professions education
