# Supplementary material for: Anesthesiologists With Advanced Degrees in Education: Qualitative Study of a Changing Paradigm
Source: JMIR Med Educ. 2022 Jun 30;8(2):e38050. doi: 10.2196/38050 (PMC9284357; doi:10.2196/38050)
Supplement: Multimedia Appendix 1 [file mededu_v8i2e38050_app1.docx]

**Interview Details**

Interview #: _______________

REDCAP ID #:______________

| **Date** | **Site** | **Interviewer** | **Participant ID (from RedCap)** | **Interview ID#**  (Interviewer Initials-Date-Number*)  *Example: MD-July 14-1)* | **Completed Study** (Y/N) |
| --- | --- | --- | --- | --- | --- |
|  |  |  |  |  |  |

**Interview Introduction:** Key points to discuss prior to the start of the interview

1. ***Introduce yourself and explain reason for the interview***
   - Hello, my name is _________ and I am here on behalf of the Pursuit of Advanced Degrees in Education for Anesthesiologists study. I’m here today to help the administrators of the study understand the potential benefits and pitfalls for anesthesiologists who have completed an advanced degree in education.
2. ***Logistics of the interview:***
   - To help the researchers understand the perspectives of anesthesiologists who have completed advanced degrees in education, we are conducting 30 minute confidential interviews to hear your experiences and opinions. Confidential means that nothing you tell me today will be linked back to you in a published format, although we will publish your institution as part of a list of sites (in aggregate) of anesthesiologists interviewed for the study. Also, we will include where the educational degrees were obtained from the panel of anesthesiologists being interviewed in aggregate, but will not link degree-conferring institutions to individual interviews.
3. ***Verbal consent:***
   - Your participation in this interview is voluntary. With your permission, we will audio and video record the interviews for transcription and thematic coding purposes only. You can stop the interview at any time and you can choose not to answer particular questions. The interview is estimated to take 25 minutes. Would you like to participate?
4. ***Demographic information:***
   - Previously collected prior to the start of the interview via REDCap.

**Interview Questions for Anesthesiologists**

| **Question Purpose** | **Questions** | **Prompting Questions**  **(Only ask if not brought up by participants based on main Qs)** | **Probes** |
| --- | --- | --- | --- |
| Introductory Questions | Thank you for filling out our pre-survey.  Tell me about your current position and what you currently do?  Please tell me about the degree you received and a brief description of it? | - What was your professional job load while getting it? | - Please go on. - Tell me more. - How did that make you feel? - And then…? - What do you mean by that? - Please explain more. - Say what you mean by […] - Why was that important to you? |
| Motivation and Timing | Please tell me about your motivation for pursuing this degree. | - What factors made you less inclined to pursue an advanced educational degree? - Did you have external pressure (e.g. from a chair or a spouse) to pursue or not to pursue the degree? | Opportunities to probe   - Timing in career - Motivating factors - Barriers to pursuing degree - External pressure to pursue |
| Perceptions of program | Please tell me about your experience pursuing this advanced degree. | - What kind of colleague would you recommend your program to? | Opportunities to probe   - Congruence between expectations and degree - Challenges in program - Time spent on the degree - Work-life balance during the degree |
| Perceptions of limitations | Please describe the limitations of the advanced degree you pursued. | - What parts of your experience did you find challenging or frustrating? - What would you change about your experience? - Were tuition costs a factor in your decision? |  |
| Attitudes and Opinions of degree | What would you tell an anesthesiologist who is interested in pursuing a higher degree in education? |  | Opportunities to probe   - Evaluating program or selecting program |
| Summation question | Is there anything that you would like to say about your experience pursuing a higher degree in education that I have not already asked? | - Probe to examine any issues mentioned - Any other people I should talk to? |  |
